# Three-dimensional computed tomography analysis of airway volume in growing class II patients treated with Frankel II appliance

**DOI:** 10.1186/s13005-024-00410-8

**Published:** 2024-02-17

**Authors:** Marwa Jameel Ahmed, Samira Diar-Bakirly, Nelson Deirs, Amar Hassan, Ahmed Ghoneima

**Affiliations:** 1 Department of Orthodontics, Hamdan Bin Mohammed College of Dental Medicine, P.O. Box 505055, Dubai, United Arab Emirates; 2 Department of Orthodontics and Oral Facial Genetics, Indiana University School of Dentistry, Indianapolis, IN USA

**Keywords:** Airway volume, Frankel appliance (FR II), 3D imaging, Class II malocclusion

## Abstract

**Objective:**

The purpose of this retrospective study was to assess the airway volume changes associated with the use of Frankel appliance (FR II) in Class II malocclusion patients using three-dimensional cone beam computed tomography (3D CBCT) imaging.

**Materials and methods:**

The sample consisted of 31 Class II malocclusion patients (mean age 9.24 ± 1.93 years old, 17 males (54.8%) and 14 females (45.2%)) treated with FR II appliance by the same orthodontist for an average of 9 months ± 20 days. CBCT images were taken before and after treatment and upper airway volume changes were measured using Dolphin 3D software version11.0 (Dolphin Imaging, Chatsworth, CA) and statistically compared.

**Results:**

Airway volume of nasal cavity, nasopharynx, oropharynx, hypopharynx and the total airway volume significantly increased after the use of FR II appliance. In addition, significant increase was reported in maxillary base, inter-molar, inter-premolar and inter-canine width. Significant increase in soft tissue thickness was only recorded opposite to CV2.

**Conclusion:**

The use of the FR II appliance in growing subjects with Class II malocclusion led to a significant increase in the upper airway volume in addition to the anticipated dental and skeletal transverse expansion effects.

## Introduction

With a global prevalence that varies between 19.56% and 23.11% in the permanent dentition and the mixed dentition, respectively, class II malocclusion has been and is still regarded as a prevalent functional and aesthetic problem [1]. Although various skeletal and dental discrepancies attribute to Class II malocclusion, yet mandibular retrognathism has been reported to be the most common feature of Class II skeletal pattern [2, 3]. Malocclusion development is frequently the result of a complex interaction between genetics and environmental variables. In addition, the soft tissue function and its effect on the craniofacial growth and development might influence skeletal development and surrounding tissues [4, 5]. Patients with class II malocclusion are reportedly more likely to experience breathing issues and have narrower airways [6, 7]. The breathing problems has been linked to a more retrusive positioned mandible, tongue and/or hyoid bone. Clinicians have long been perplexed by the question of whether the retrusive mandible is the source of the reduced airway volume or whether the change in head posture caused by mouth breathing is the cause of the retrusive mandible.

When airway volume was compared in Class I and Class II malocclusion patients, some studies reported a higher risk of developing breathing problems and diminished airway volume specifically related to Class II individuals [6–12]. The use of functional appliances to improve the mandibular position by bringing it forward and consequently increasing the airway volume has been reported in several studies [13–18]. Removable functional appliances as activators, bionators and twin block reportedly increased airway volumes at different anatomical sites such as the nasopharynx, oropharynx, and hypopharynx [8, 19]. However, the reliability of these reports has always been questioned because most of the studies assessed the airway volume using cephalometric measurements (2D imaging) rather than 3D imaging tools such as cone beam computed tomography (CBCT).

Frankel appliance (FR II) is a tissue borne functional appliance used for treating Class II patients with retrognathic mandible by actively positioning the mandible forward. Its effect on the mandible ranges from minimal change in mandibular length to significant increase [20–25]. The aim of this retrospective study was to assess the airway volume changes associated with the use of FR II in Class II malocclusion patients using 3D CBCT imaging.

## Materials and methods

This retrospective study was performed using a sample of 31 CBCT images of pre-treatment and post-treatment records for orthodontic patients. Records were collected from one private practice and were treated by the same orthodontist. The subjects’ mean age was 9.24 ± 1.93 years old, 17 males (54.8%) and 14 females (45.2%), classified as Caucasians with Class II malocclusion according to Angle’s classification. All patients were treated with FR II for an average of 9 months (mean age after treatment 9.98 ± 1.91 years). Patients with craniofacial abnormalities, traumatic injury, systematic diseases, TMJ abnormalities, reportedly airway abnormalities, pharyngeal and/or nasal disease, subjects on medications, previous orthodontic and/or surgical treatment, and low-quality CBCT scans with artefacts were excluded from the sample. Absence of sinus infection or obstruction was confirmed by checking the radiographic density of the sinuses with coronal slices prior to the measurement.

All CBCTs were taken with i-CAT CBCT (Imaging Sciences, Hatfield, PA) set for full 13 cm field of view, 20 s of scanning time, and a resolution of 0.3 mm voxel size. The CBCT were taken for each patient in DICOM format (Digital Imaging and Communications in Medicine), then coded and randomly analyzed by the primary investigator who was blinded to the code. Blinding was intended to eliminate bias and enhance the objectivity of the study. Analysis of upper airway volume was performed on the 3D CBCT images. Analysis of soft tissue thickness of the upper airway was performed on the sagittal sections of the CBCT images, while maxillary base width, inter-canine width, inter-premolar width, and inter-molar width were measured on the coronal sections of the CBCT images. The selected parameters were measured using Dolphin 3D software, version 11.0 (Dolphin Imaging, Chatsworth, CA). Changes between the before and after 3D measurements were recorded for the 31 subjects. Dental occlusion was confirmed for each side for all subjects using the CBCT volumetric rendering. The prevertebral soft tissue thickness was measured at different levels along the airway.

**Fig. 1:**
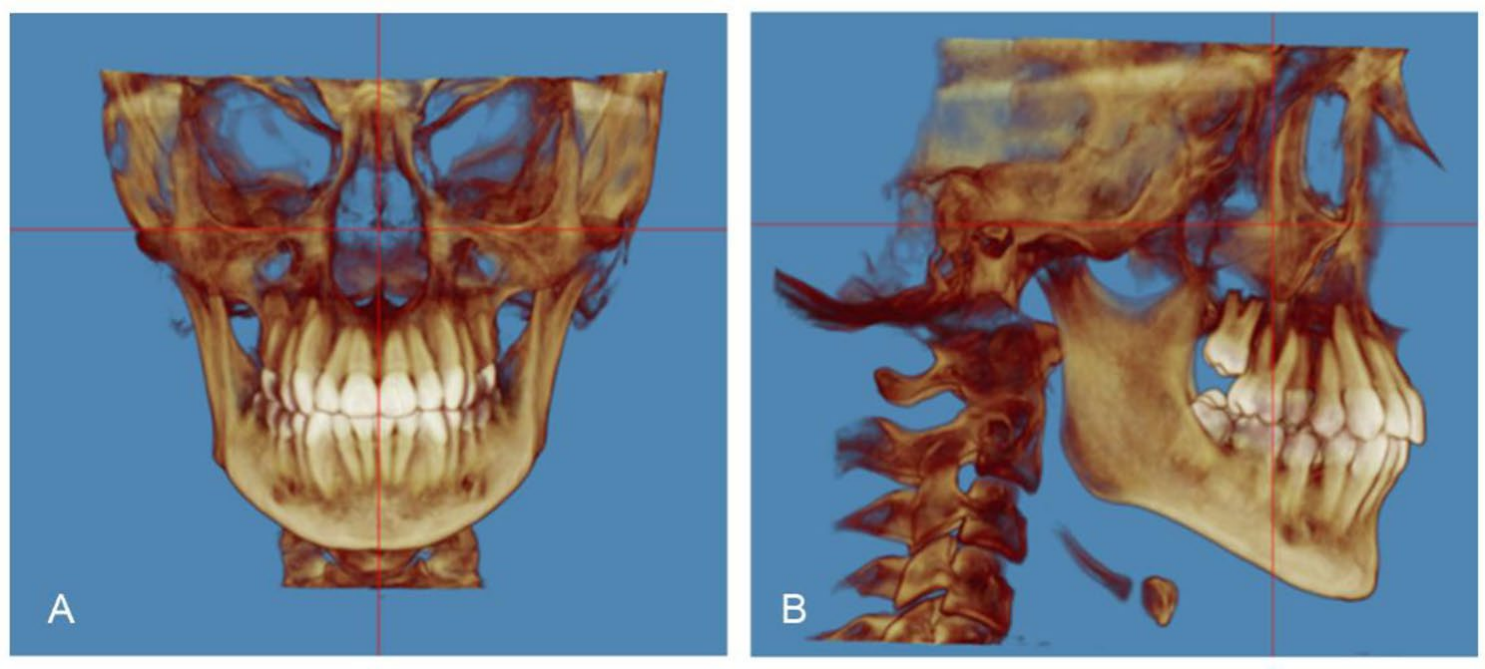
CBCT image orientations **(A)** Midsagittal plane **(B)** Axial and coronal plane

**Fig. 2:**
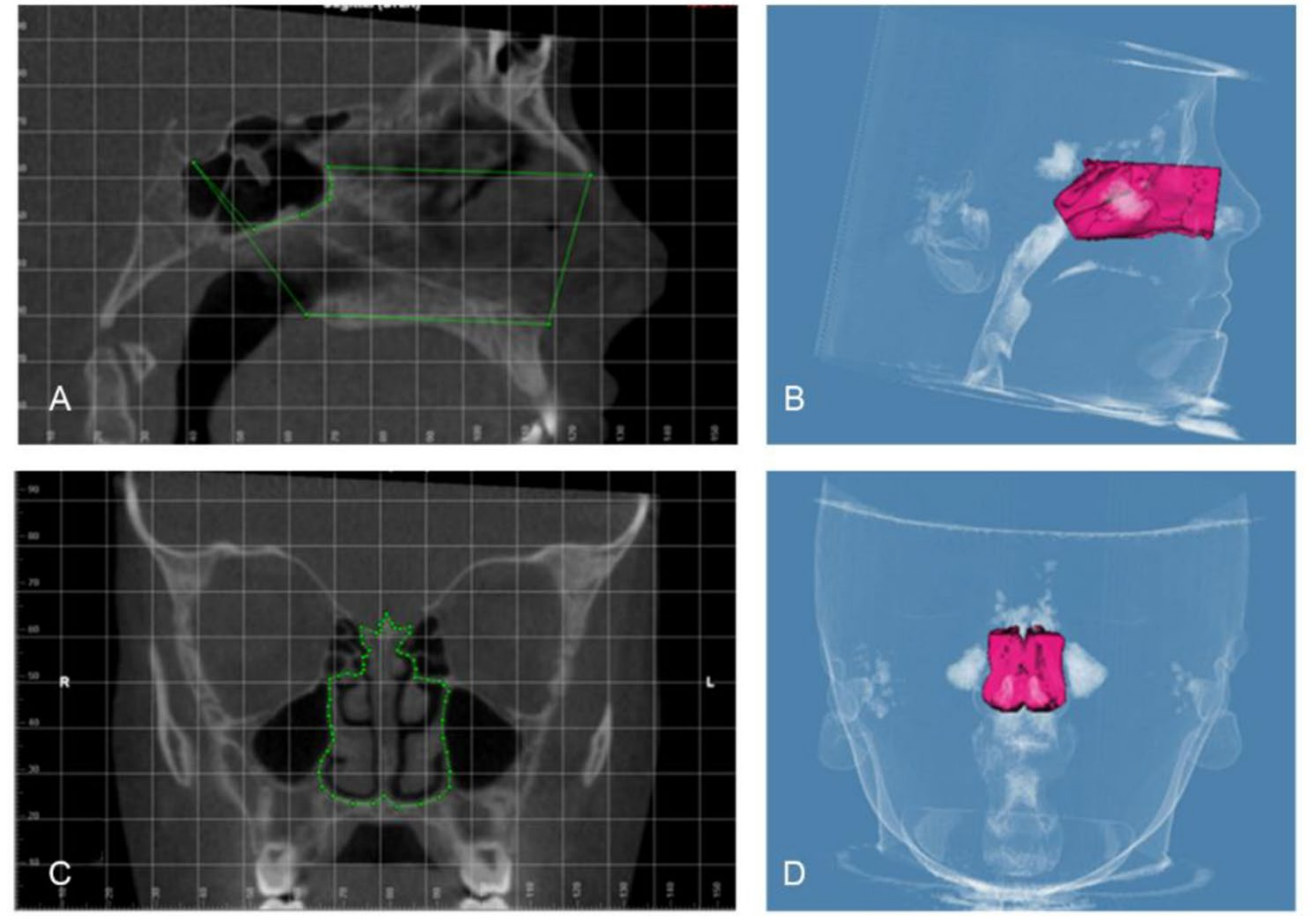
Nasal cavity airway boundaries **(A)** sagittal plane **(B)** Side view of the nasal cavity airway volume **(C)** coronal plane **(D)** frontal view of the nasal cavity airway volume

**Fig. 3:**
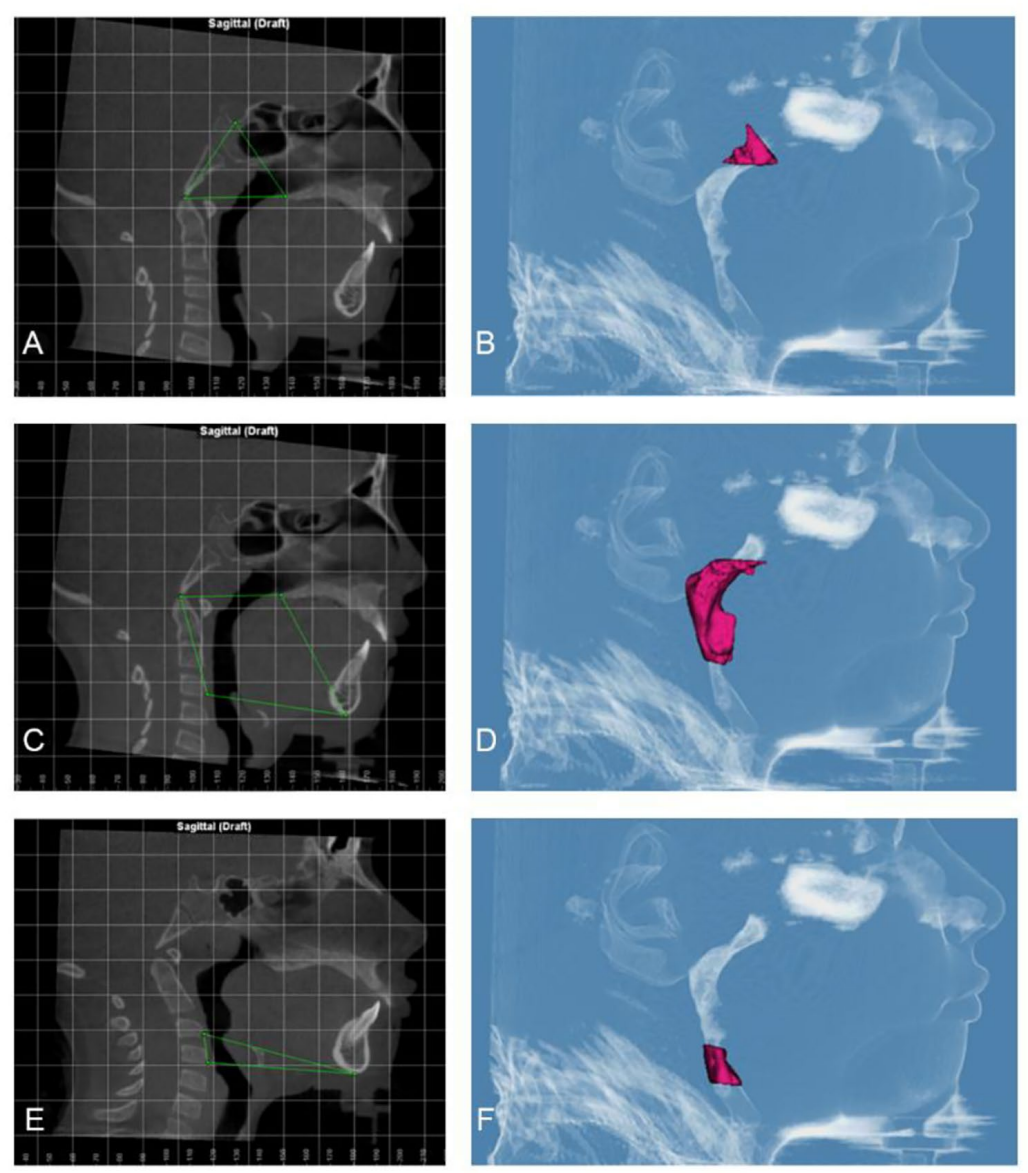
**(A)** Sagittal view of the nasopharynx airway boundaries **(B)** Side view of the nasopharynx airway volume **(C)** Sagittal view of the oropharynx airway boundaries **(D)** Side view of the oropharynx airway volume **(E)** Sagittal view of the hypopharynx airway boundaries **(F)** Side view of the hypopharynx airway volume

**Fig. 4:**
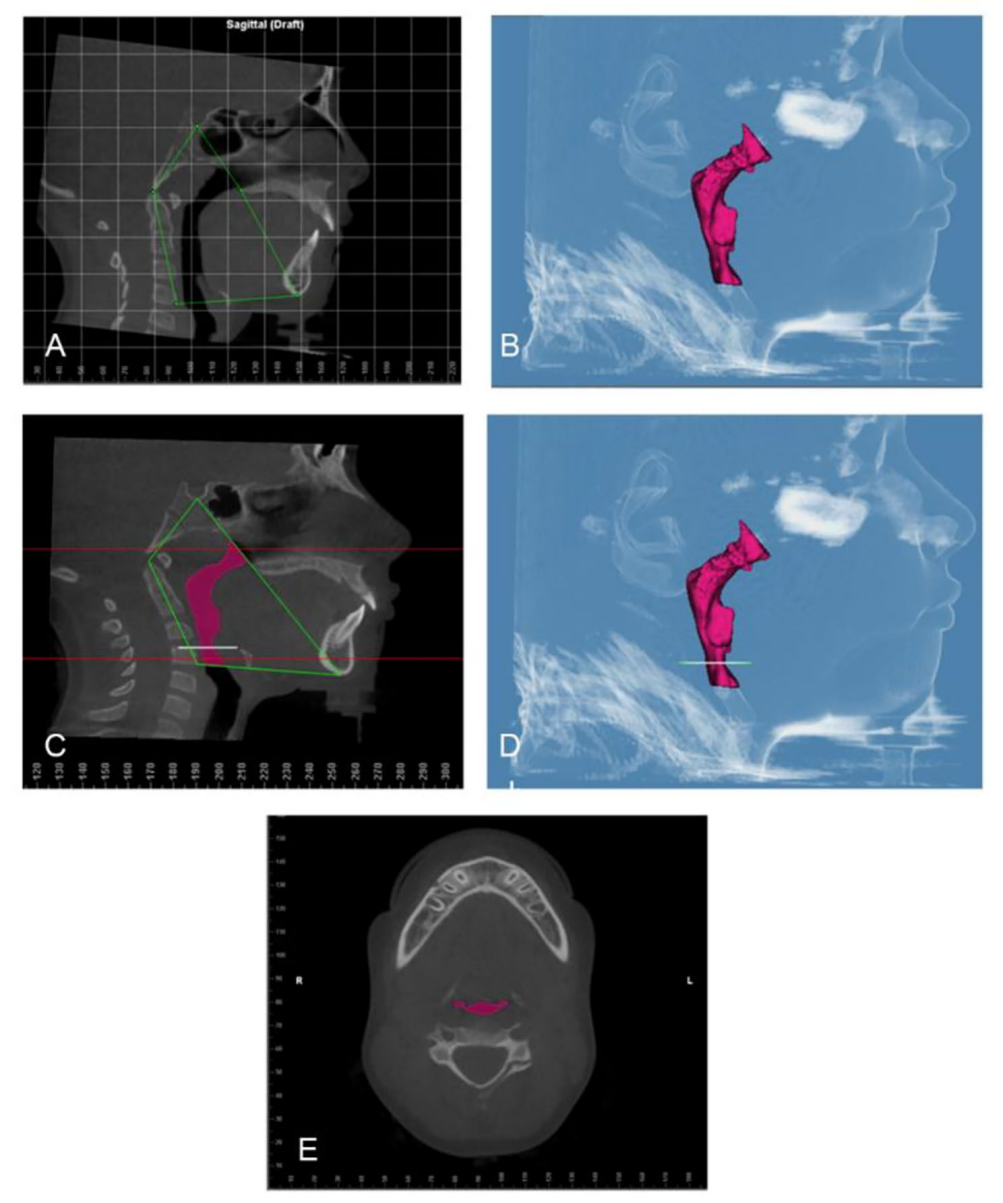
**(A)** Sagittal view of the total airway boundaries **(B)** Side view of the total airway volume **(C)** Sagittal view of the MCA **(D)** MCA with total airway volume **(E)** Axial view of the MCA

**Fig. 5:**
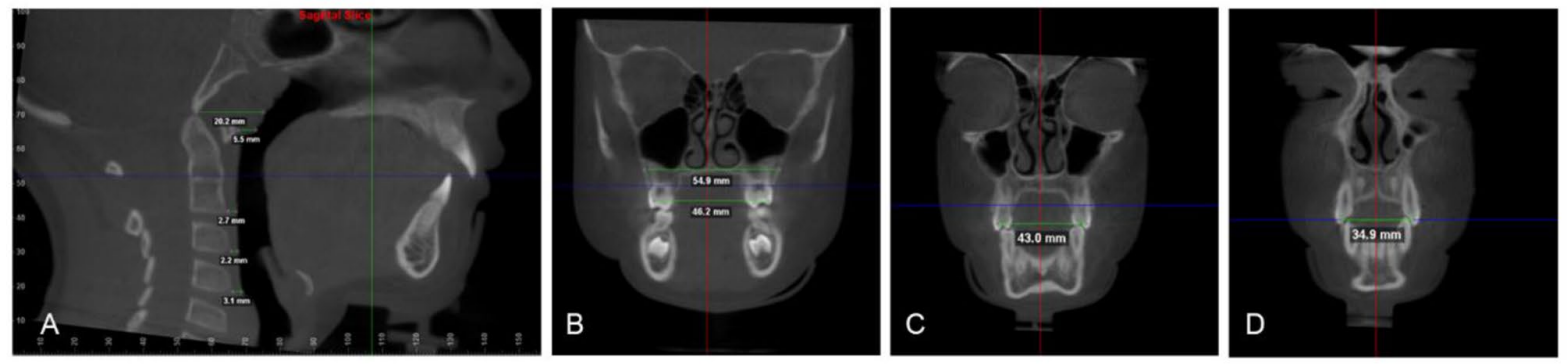
**(A)** Prevertebral soft tissue thickness **(B)** Maxillary base and inter-molar width **(C)** Inter-premolar width **(D)** Inter-canine width

The airway volume and the most constricted area (MCA) were evaluated using the same software on the 3D images with a threshold level of 50% (sensitivity level) to detect airway space [26]. The 3D volumetric images were oriented in Dolphin imaging software as follows: the midsagittal plane aligned to the midline of the face passing through soft tissue nasion and subnasale point (Fig. 1A), the axial plane aligned to the level of Frankfurt horizontal (FH) plane (Po-Or) parallel to the floor, and the coronal plane aligned to the level of the furcation point of the right maxillary first molar (Fig. 1B). The boundaries of each airway segment, the MCA, the prevertebral soft tissue thickness, dental arch width and maxillary base width are defined in (Table 1; Figs. 2, 3, 4 and 5). Intra-examiner reliability test was performed by repeating all measurements for 10 cases with 2 weeks interval in-between.

**Table 1:** Definition of parameters used in the study

Parameter	Plane	Boundaries
The nasal cavity (Fig. 2)	Sagittal Plane Coronal Plane	Anterior: by the line connecting the anterior nasal spine (ANS) – the tip of the nasal bone –Nasion (N) Posterior: the line extending from floor Sella (S) – Posterior nasal spine (PNS) Superior: the line connecting the N – S Inferior: the line extending from ANS –PNS the outline of the nasal cavity in a section including the maxillary first molar bifurcations area starting at the crista galli, running downward toward the nasal floor and passing through the sidewalls of the right and left nasal cavity
The nasopharynx (Fig. 3A, B)	Sagittal Plane	Anterior: the line extending from S –PNS Posterior: the line extending from S – tip of the odontoid process Inferior: the line extending from PNS – tip of the odontoid process
The oropharynx (Fig. 3C, D)	Sagittal Plane	Anterior: the line extending from PNS – symphysis of the mandible Posterior: the line extending from the tip of the odontoid process – anterior-inferior border of C3 Superior: the line extending from PNS – tip of the odontoid process Inferior: the line extending from the anterior -inferior border of C3- symphysis of the mandible
The hypopharynx (Fig. 3E, F)	Sagittal Plane	Anterior: symphysis of the mandible Posterior: the line extending from the anterior-inferior border of C3– anterior-inferior border of C4 Superior: the line extending from the anterior -inferior border of C3- symphysis of the mandible Inferior: the line extending from the anterior -inferior border of C4- symphysis of the mandible
Total airway (Fig. 4A, B) and the boundary to detect the most constricted area of the airway (Fig. 4C, D, E)	Sagittal Plane	Anterior: the line extending from S –PNS – symphysis of mandible Posterior: the line extending from S - the tip of the odontoid process – anterior-inferior border of C4 Inferior: the line extending from the anterior-inferior border of C4- symphysis of the mandible
Soft tissue thickness opposite to Basion (Ba) (Fig. 5A)	Sagittal Plane	The prevertebral soft tissue thickness as a distance parallel to FH plane from the most infero-posterior point on the anterior rim of foramen magnum :Basion (Ba)
Soft tissue thickness opposite to Atlas (Fig. 5A)	Sagittal Plane	The prevertebral soft tissue thickness as a distance parallel to FH plane from the most anterior point on the anterior arch of the atlas vertebrae (AA)
Soft tissue thickness opposite to CV2 (Fig. 5A)	Sagittal Plane	The prevertebral soft tissue thickness as a distance parallel to FH plane from the most inferior-anterior point of CV2 (CV2ia)
Soft tissue thickness opposite to CV3 (Fig. 5A)	Sagittal Plane	The prevertebral soft tissue thickness as a distance parallel to FH plane from the most inferior-anterior point of CV3 (CV3ia)
Soft tissue thickness opposite to CV4 (Fig. 5A)	Sagittal Plane	The prevertebral soft tissue thickness as distance parallel to FH plane from the most inferior-anterior point of CV4 (CV4ia)
Inter-molar (Fig. 5B)	Coronal Plane	Distance between the disto-buccal cusps of right and left first molars, parallel to FH plane
Inter-premolar (Fig. 5C)	Coronal Plane	Distance between the disto-buccal cusps of right and left first pre-molars, parallel to FH plane
Inter-canine (Fig. 5D)	Coronal Plane	Distance between the cusp Tips of right and left canines, parallel to FH plane
Maxillary width (Fig. 5B)	Coronal Plane	The point located at the depth of concavity of the lateral maxillary contour, at the junction of the maxilla and the zygomatic buttress, parallel to FH plane

### Statistical analysis

Data was analyzed using IBM-SPSS for windows version 23.0 (SPSS Inc., Chicago, IL). Measures of percentage, tendency and dispersion were performed as descriptive statistics for categorical and continuous data respectively. Kolmogorov-Smirnov was used to test the normality of continuous variables. Paired t-test was used to compare means between before and after continuous data. Correlation coefficient was used to test the linear correlation between variables. ANOVA test was used to compare means of continuous data between classes. Internal consistency and agreement were tested by using Bland-Altman methods. *P*-value ≤ 0.05 was considered significant.

## Results

The intra-examiner reliability test showed no statistically significant differences between readings and excellent intra-examiner reliability (intra-class correlation coefficient ≥ 0.08) for all measurements. Comparison between before and after FR II treatment showed statistically significant increase in airway volume measurements in the following parameters: nasal cavity (*P* = 0.000), nasopharynx (*P* = 0.004), oropharynx (*P* = 0.002), hypopharynx (*P* = 0.001), and total airway (*P* = 0.001). MCA measurement showed statistically significant increase after FR II treatment (*P* = 0.005) (Table 2). Soft tissue thickness at CV2 showed statistically significant increase with a *P*-value of 0.035, while no significant changes were found for the soft tissue thickness at Basion, atlas, CV3 and CV4 (Table 3). Inter-maxillary width, inter-molar, inter-premolar, and inter-canine width all showed statistically significant increase with FR II treatment (*P* = 0.000) (Table 4).

**Table 2:** Airway volume and MCA measurements

Parameters	Before treatment				After treatment					Change				95% Confidence Interval of the Difference		ICC	*P*-value
	Mean	SD	Min	Max	Mean	SD	Min	Max	Mean		SD	SE	Lower		Upper		
Nasal Cavity airway volume mm^3^	16942.0	3992.5	9282.0	24494.0	21285.0	4685.1	11577.0	32033.0	4343.0		4440.5	797.5	2714.2		5971.8	0.98	0.000*
Nasopharynx airway volume mm^3^	4756.3	3992.5	9282.0	24494.0	5879.8	2372.2	2539.0	12626.0	1123.5		1902.0	341.6	425.9		1821.2	0.99	0.004*
Oropharynx airway volume mm^3^	14659.2	6281.3	5797.0	29606.0	29356.8	56545.0	7292.0	331781.0	14697.6		56620.1	10169.3	-6070.8		35466.1	1.00	0.002*
Hypopharynx airway volume mm^3^	3427.2	1748.9	1384.0	8555.0	4662.2	1841.8	1731.0	8832.0	1455.8		1821.8	388.4	648.1		2263.6	1.00	0.001*
Total airway volume mm^3^	22722.8	8572.7	9063.0	43878.0	30254.4	9310.6	17367.0	52707.0	8471.0		9103.2	1858.2	4627.1		12315.0	1.00	0.001*
MCA mm^2^	158.7	74.8	30.0	373.0	222.2	111.2	74.0	494.0	63.6		102.6	18.7	25.2		101.9	0.99	0.005*

* Significant at *P* ≤ 0.05

**Table 3:** Soft tissue thickness measurements

Parameters	Before treatment				After treatment				Change			95% Confidence Interval of the Difference		ICC	*P*-value
	Mean	SD	Min	Max	Mean	SD	Min	Max	Mean	SD	SE	Lower	Upper		
Soft tissue thickness at basion	20.4	5.4	13.4	35.6	20.7	5.0	13.1	33.8	0.3	5.1	0.9	-1.5	2.2	0.986	0.572
Soft tissue thickness at atlas	5.4	4.1	1.8	23.8	5.3	3.9	1.6	21.7	-0.1	2.5	0.5	-1.0	0.9	0.987	0.877
Soft tissue thickness at CV2	3.4	0.7	1.9	4.6	3.7	0.7	2.3	5.4	0.3	0.7	0.1	0.0	0.5	0.889	0.035*
Soft tissue thickness at CV3	3.4	1.0	2.0	7.1	3.7	0.8	2.6	5.7	0.3	1.1	0.2	-0.1	0.7	0.877	0.054
Soft tissue thickness at CV4	5.8	3.0	2.5	12.5	5.1	2.2	3.1	11.7	-0.6	2.8	0.5	-1.7	0.4	0.931	0.347

* Significant at *P* ≤ 0.05

**Table 4:** Maxillary base, inter-molar, inter-premolar and inter-canine width measurements

Parameters	Before treatment				After treatment				Change			95% Confidence interval of the difference		ICC	*P*-value
	Mean	SD	Min	Max	Mean	SD	Min	Max	Mean	SD	SE	Lower	Upper		
Maxillary width	62.4	3.4	54.9	67.5	64.7	3.4	59.3	72.0	2.3	2.3	0.4	1.4	3.1	0.823	0.000*
Inter-molar width	52.2	3.3	46.2	59.9	56.2	2.9	51.7	65.6	3.9	3.1	0.6	2.8	5.1	0.979	0.000*
Inter-premolar width	41.3	4.2	29.8	48.7	46.2	2.1	42.5	49.5	4.9	3.7	0.7	3.5	6.3	0.955	0.000*
Inter-canine width	33.6	2.9	26.5	39.2	38.4	2.0	33.1	42.7	4.6	2.7	0.5	3.5	5.7	0.927	0.000*

*Significant at *P* ≤ 0.05

## Discussion

Breathing pattern and upper airway volume significantly affect the facial growth and craniofacial development. Therefore, patent airway is essential in developing normal breathing and craniofacial development. Children with Class II malocclusion and retrusive mandible are at a higher risk of breathing problems due to reduced airway dimensions [6, 7]. It seemed prudent to examine the advantage of expanding the airway volume after moving the jaw forward given the impact of functional appliances on mandibular posture through the effect on the masticatory and facial muscles [27]. In this study, we used 3D CBCT imaging to assess the impact of FR II on airway dimensions. We examined the upper airway capacity for each segment independently to determine whether the appliance has any different effects on different areas. The choice of using the scans obtained from a low-dose CBCT machine was justified by its superior diagnostic capabilities and the inability of alternative imaging methods to provide the necessary information related to airway dimensions.

There is a consensus in the literature that cephalometric radiographs can be used to detect linear and angular changes of skeletal, dental, and soft tissue relations whether with age or after orthopaedic or orthodontic treatment, in addition to being a primary screening tool for volumetric changes [28]. Yet, using 2D imaging, small volumetric changes might be missed especially when dealing with 3D restricted complex volumes [29, 30]. Several studies contended the use and reliability of cephalometric radiographs when measuring the nasopharyngeal space, because it is considered a complex space to measure, especially when it comes to the most restricted volumes [28–32].

Previous reports, using 2D cephalometric analysis, indicated an increase in nasopharynx and hypopharynx airway dimensions after the use of FR II appliance [33, 34]. The current study, using 3D CBCT, confirm these results and supports the increase in the airway capacity in the nasopharynx area linked with using the FR II appliance. This increase however, has been inconsistently reported with the use of other functional appliances, such as the activator, bionator and X bow [35, 36]. One study reported that the nasopharynx airway dimensions did not significantly increase with the twin block functional appliances when measured by cephalometric 2D imaging yet, the same appliance led to increase in airway volume at the nasopharynx when measured by the 3D CBCT imaging tool [37].

The oropharynx airway volume, in the current study, significantly increased with the use of FR II appliance. This increase could be explained by the forward repositioning of the tongue and soft palate after mandibular advancement [38, 39]. Forward horizontal pull of the hyoid bone by the action of muscles attached to the mandible or the tongue could be another postulated explanation behind the increased oropharyngeal space. The hypopharynx airway volume significantly increased with the use of FR II appliance as well, which could also be related to the forward positioning of the mandible and horizontal movement of the hyoid bone.

Soft tissue thickness of the posterior pharyngeal wall opposite to Basion, atlas, CV3 and CV4 did not show significant change from before and after use of FR II appliance, thus ruling out any role of the soft tissue to the changes in the hypopharyngeral airway volume measurements. The only significant increase in soft tissue thickness was reported opposite to CV2.

The findings of the current study were in line with previous reports of the significant increase in maxillary base width, intermolar, inter-premolar and inter-canine width [40–42]. The increase in the maxillary width is expected to contribute to the increase in the airway volume at the nasal cavity level. From a clinical point of view, orthodontic treatment using FRII for Class II malocclusion can potentially lead to improving respiratory function and reducing the risk of sleep-disordered breathing. However, orthodontic decisions should be tailored to address specific patient needs, considering all influential factors such as facial aesthetics, occlusion, and potential airway concerns.

It is important to note that while 3D CBCT scans are useful and reliable tool for estimating airway volume, they only provide a snapshot of the locations of soft tissues during a transient phase of the breathing cycle. Breathing is a complex multivariable function that cannot be described by airway dimensions only, in the current study, due to the retrospective nature, no functional breathing assessment was performed. The absence of a functional breathing assessment is a limitation that emphasizes the need for caution when generalizing the findings.

## Conclusion

FR II appliance therapy is associated with significant increase in upper airway volume and the MCA of the airway. FR II appliance also causes dental expansion as well as expansion in the maxillary base.

## Data Availability

The datasets used and/or analyzed for the current study are available from the corresponding authors on reasonable request.
